# Innovation and achievement for primary care in Brazil: new challenges

**DOI:** 10.3399/bjgpopen17X100857

**Published:** 2017-04-19

**Authors:** Airton Tetelbom Stein, Cleusa Pinheiro Ferri

**Affiliations:** 1 GP & professor, Public Health Department, Universidade Federal de Ciências da Saúde de Porto Alegre (UFCSPA), Porto Alegre, Brazil; 2 HTA Coordinator, Conceição Hospital, Porto Alegre, Brazil; 3 Affiliated Professor, Psychobiology, Universidade Federal de Sao Paulo, Sao Paulo, Brazil; 4 Senior Epidemiologist, Institute of Health Education and Science, Hospital Alemao Oswaldo Cruz, Sao Paulo, Brazil

**Keywords:** primary health care, innovation, Brazil

Brazil occupies half of the South American landmass, and is the fifth largest country in the world. The current population estimate is 207 million.^1^ Demographic and epidemiological changes, as well as nutritional transition, have affected mortality and morbidity in the country. The leading causes of disability-adjusted life years (DALYs) in 2010 were ischaemic heart disease, interpersonal violence, lower back pain, stroke, and road injury.^2^

## Community empowerment and coverage

The main principles of the Brazilian National Health System (SUS), are universal access, comprehensive care, and equity of actions. The SUS model has emphasised the rapid scaling-up of community-based care in order to provide comprehensive primary health care (PHC). Among a number of important initiatives undertaken to develop this approach are the introduction of lay health workers (*agentes comunitários de saúde*), and an increased focus on community empowerment. These are both examples of important innovations in the public health system.

The PHC has increased its coverage around the country, initially through the Community Health Agents Programme (*Programa Agentes Comunitários de Saúde*) and subsequently by the Family Health Strategy (FHS) (*Estratégia de Saúde da Família*), introduced in the 1990s. This has focused on the reorganisation of the care provided to families and communities in specific territories, and the integration of general practice into the system to promote public health actions, and provide medical care.^3^

There are 41 000 FHS teams across the country, covering 64% of the entire Brazilian population, and providing care for more than 120 million inhabitants.^3^ Each team comprises GPs, nurses, auxiliary nurses, and lay health workers, focusing on a robust interdisciplinary approach to provide primary care for a defined population. There is an increasing amount of evidence for its role in improving access and health outcomes for the Brazilian population. Two studies have shown improved access, wider use, and increased satisfaction with the FHS.^4,5^ Another study has shown improved equity in healthcare utilisation,^6^ and there have also been studies showing better health outcomes among Brazilians covered by the FHS.^7,8^

Another programme introduced at the national level is the More Doctors programme (*Programa Mais Médicos*), which is supported by the federal government with the aim of strengthening PHC in the country. This programme provides more physicians to less resourced regions, promoted the development of medical schools in areas where they did not previously exist, and increased vocational training in general practice.

## Technology

A number of public telehealth initiatives have been implemented to provide better access to higher quality care, and allow referrals to be made more easily within the system.^9,10^ Information technology, as a way to support and improve the diagnosis and management of conditions that can be dealt with at the primary care level, is essential in order to have a more effective health system, and is one of the greatest challenges in terms of innovation for PHC throughout the country. Studies of its effectiveness using robust methodology are required in order to ensure it is being used in the best possible ways.

The Brazilian Ministry of Health has recently produced guidance for health technology assessment for PHC in order to identify the types and characteristics of studies that should be developed to improve decision making at this level of care, such as the rapid appraisal of new technologies, the use of systematic reviews, and evaluation of budget impact. This guidance is in press and will be published in 2017.^11^

## Challenges

The health system faces the challenge of implementing quality PHC throughout a large country with many socioeconomic differences and serious inequities in access to health care. The number of health professionals, including GPs, who have adequate qualification, are insufficient to provide universal coverage in every part of the country. The challenge also includes the delivery of effective interventions in remote areas, which are culturally and socially sensitive, and the coordination of long-term care between the primary and specialised care sectors, a challenge that is increased by the rapid ageing of the Brazilian population.

There has been an increase in PHC investment, but it is still insufficient. Programmes, such as the FHS, appear to be cost-effective; however, due to the current economic and political situation in the country, the achievements in PHC, and the remarkable health system reform in Brazil over the last two decades, are under threat. Strengthening PHC in Brazil, through these and other initiatives, is essential to guarantee the SUS principles of universal access, comprehensive care, and healthcare equity.

**Figure 1.**
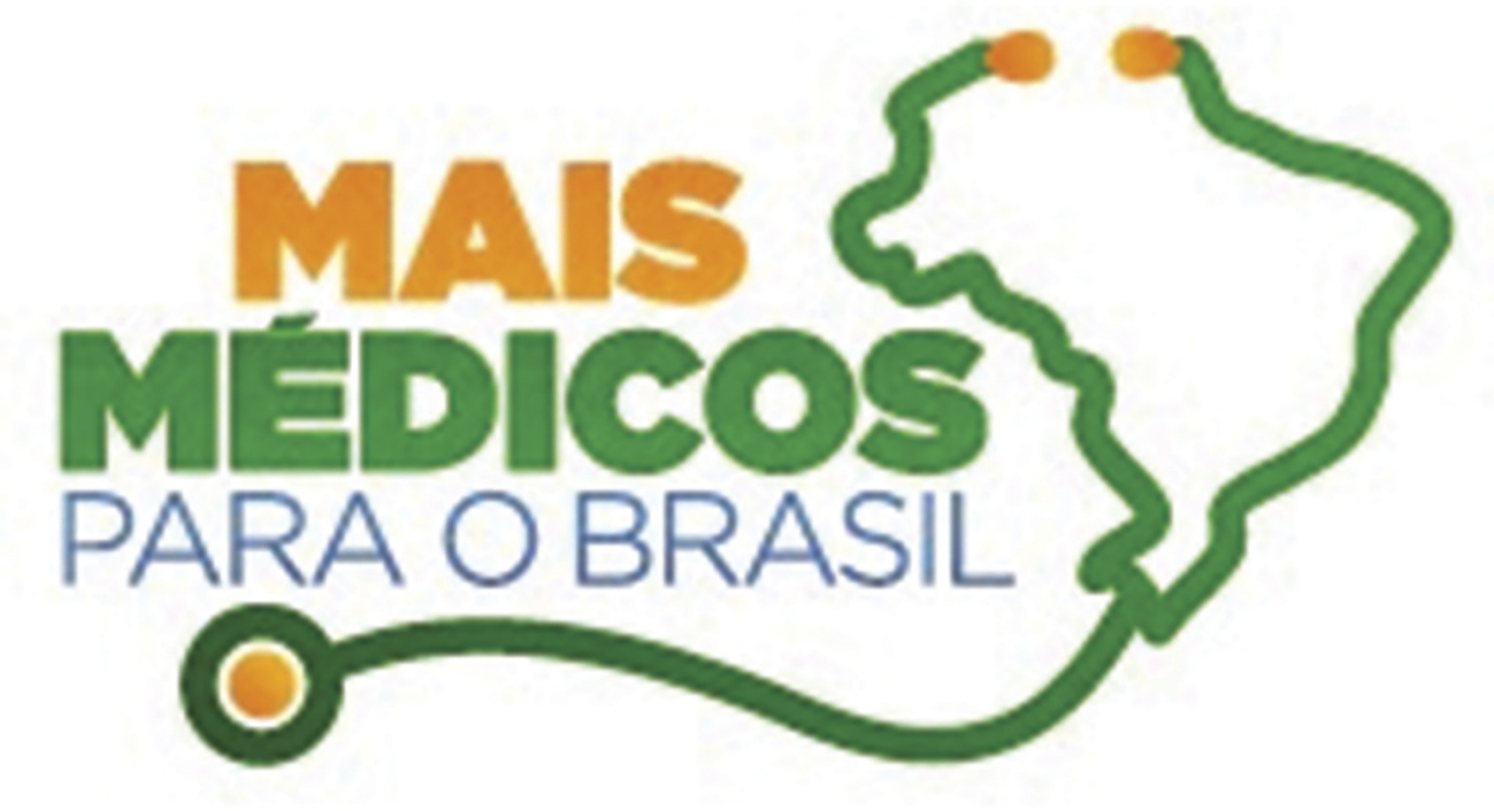
More Doctors Programme
